# Circular RNA circREPS2 Acts as a Sponge of miR-558 to Suppress Gastric Cancer Progression by Regulating RUNX3/β-catenin Signaling

**DOI:** 10.1016/j.omtn.2020.06.026

**Published:** 2020-06-27

**Authors:** Xiong Guo, Xinglong Dai, Jianjun Liu, Anqi Cheng, Chuan Qin, Ziwei Wang

**Affiliations:** 1 Department of Gastrointestinal Surgery, Laboratory Research Center, The First Affiliated Hospital of Chongqing Medical University, Chongqing 400010, P.R. China; 2 Department of Gastrointestinal Surgery, Three Gorges Hospital, Chongqing University, Chongqing 404000, P.R. China

**Keywords:** circREPS2, gastric cancer, miR-558, RUNX3, EMT

## Abstract

Circular RNAs (circRNAs) play an essential regulatory role in multiple cancers. However, the role of a large number of circRNAs in gastric cancer (GC) is still unknown. Here, hsa_circ_0139996 (circREPS2), a novel circRNA that was significantly downregulated in GC, was selected for further investigation. circREPS2 was validated and analyzed by DNA sequencing and quantitative real-time PCR. The roles of circREPS2 in GC cells were verified by gain- and loss-of-function experiments. Bioinformatics analysis, luciferase reporter, RNA pull-down, and RNA immunoprecipitation assays were performed to evaluate the functional mechanism of circREPS2 on microRNA-558 (miR-558)/RUNX3/β-catenin axis in GC cells. In the present study, we found that circREPS2 was downregulated in GC tissues and cell lines. Low expression of circREPS2 was associated with a higher tumor-node-metastasis (TNM) stage, poor tumor differentiation, and larger tumor size in GC patients. Functionally, circREPS2 significantly inhibited GC cell proliferation, migration, invasion, and epithelial-mesenchymal transformation (EMT) *in vitro* and tumorigenesis *in vivo*. Furthermore, our data demonstrated that circREPS2 acted as a miR-558 sponge and upregulated RUNX3 expression to inactivate β-catenin signaling in GC cells. In conclusion, circREPS2 suppresses the progression of GC via miR-558/RUNX3/β-catenin signaling and is a novel promising biomarker and target for GC treatment.

## Graphical Abstract

**Figure:**
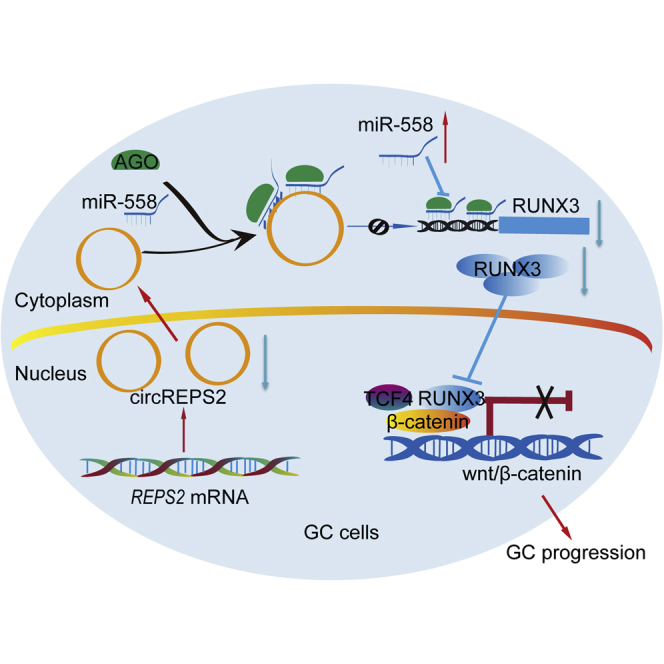

## Teaser

Wang and colleagues identified a novel circular RNA, circREPS2, which is dysregulated in GC. circREPS2 upregulates RUNX3 expression by sponging miR-558, leading to inhibition of β-catenin pathway transcriptional activity, thereby inhibiting GC cell proliferation, metastasis, and EMT. These observations suggest that circREPS2 may serve as a therapeutic target for GC.

## Introduction

Gastric cancer (GC) is one of the epidemic tumors of the digestive system not only in China but also in the world.^1^ It is the fifth most frequently diagnosed cancer and the third leading cause of cancer-related deaths.^2^ Due to advances in many diagnostic methods and surgical procedures, the morbidity and mortality of GC have been stably decreasing in the last few years.^3^ However, because of the aggressiveness and recurrence of the tumor, the overall survival (OS) of GC patients within 5 years is low at less than 29%.^4^ Therefore, there is an urgent need to explore molecular patterns and find new therapies for GC, especially for complicated gene regulation axes or networks.

Circular RNAs (circRNAs), comprising a relatively large family of noncoding RNAs, originate from noncanonical splicing events called back-splicing.^5^^,^^6^ circRNAs have become ideal biomarkers for multiple tumors and have been shown to exert suitable potential therapeutic effects on cancers.^7^ To date, most circRNAs have been identified and proposed to act as microRNA (miRNA) sponges.^8^ Cdr1as is a well-known circRNA that contains more than seventy binding sites and serves as a sponge for miRNA-7 in neuronal tissues.^8^ Additionally, circRNAs can function through related proteins. circFndc3b interacts with the RNA-binding protein FUS to regulate VEGF, thus promoting cardiac repair after myocardial injury.^9^ In addition, circRNAs can be translated. Xia et al.^10^ reported that circular RNA AKT3 could encode a novel 174 amino acid (aa) protein, called AKT3-174aa, which inhibits the tumorigenesis of glioblastoma. In short, circRNAs are involved in multiple disease types and play roles in gene expression, proliferation, apoptosis, autophagy, EMT, etc.^11^ However, a large number of circRNAs in GC are still unknown, and further studies are needed to understand their functional mechanisms.

In our study, we used human circRNA microarray analysis to identify differentially expressed circRNAs in GC tissues. We identified hsa_circ_0139996 (termed as circREPS2), derived from the RALBP1-associated Eps domain containing 2 (REPS2) gene, that was stably downregulated in GC tissue samples and cell lines. Importantly, we found that circREPS2 exerts its inhibitory effect in GC cells by sponging oncogenic miR-558 and upregulating the expression level of RUNX3 to inactivate β-catenin signaling. Therefore, circREPS2 can be a promising biomarker and therapeutic target for GC patients.

## Results

### Validation, Expression, and Characterization of circREPS2 in GC Tissues and Cell Lines

**Figure 1:**
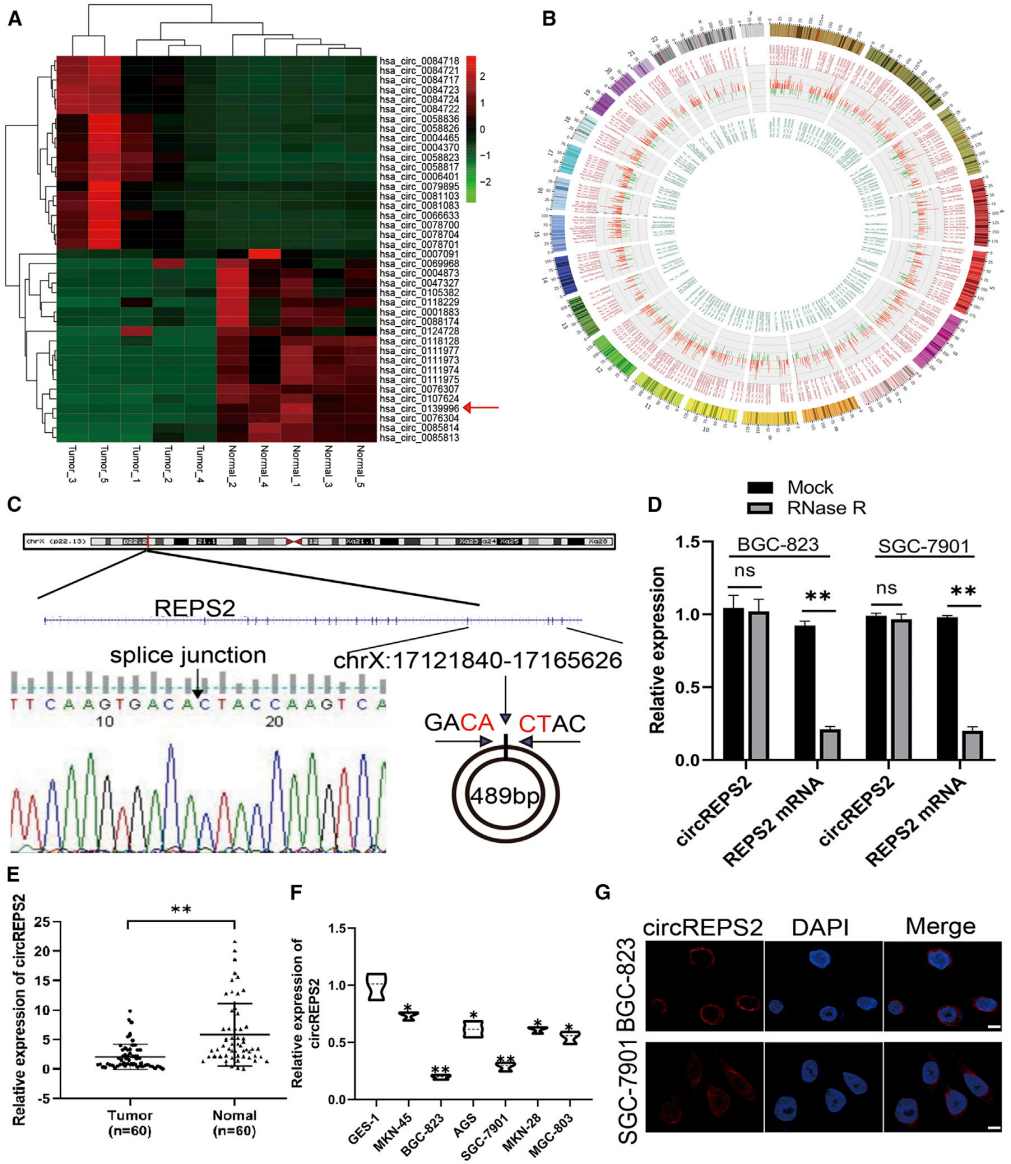
Validation, Expression, and Characterization of circREPS2 in GC Tissues and Cell Lines (A) Cluster heatmap of top 20 up- and downregulated differentially expressed circRNAs. (B) Circos plots of the differentially expressed circRNAs in GC tissues. Outer, upregulated circRNAs (red). Inner, downregulated circRNAs (green). (C) The head-to-tail splicing of circREPS2 was confirmed by Sanger sequencing. (D) Quantitative real-time PCR analysis of the expression of circREPS2 and REPS2 mRNA in the presence or absence of RNase R in BGC-823 and SGC-7901 cell lines. (E) Quantitative real-time PCR analysis of the expression of circREPS2 in 60 paired GC tissues and adjacent normal tissues. (F) Quantitative real-time PCR analysis of the expression of circREPS2 in various human GC cell lines (BGC-823, AGS, MKN-45, MGC-803, MKN-28, and SGC-7901) and a human gastric epithelial cell line (GES-1). (G) FISH analysis of the cellular localization of circREPS2 in BGC-823 and SGC-7901 cells. Nuclei were stained with DAPI (scale bar, 10 μm). Values are shown as the mean ± standard error of the mean based on three independent experiments. ∗p < 0.05, ∗∗p < 0.01.

The microarray expression profile comparing circRNAs in five paired GC tissues and noncancerous tissues has been described in our previously study.^12^ In fact, 143,527 circRNAs were detected and a total of 5,508 circRNAs (filtered by |FC (fold change)| ≥ 2 and p < 0.05) were differentially expressed between the GC tissues and paired adjacent non-tumor tissues. The heatmap of top 20 up and downregulated circRNAs shown in (Figure 1A), and we found that circREPS2 is one of the most significant downregulated circRNAs in GC. Notably, approximately 70%–80% of circRNAs were derived from their parental genes and were located in diverse genomic regions (Figure 1B). Then, we confirmed circREPS2 (located at chrX: 17121840–17165626) is derived from parental gene REPS2, which is located on chromosome Xp22.2 according to the human reference genome (GRCh37/hg19). Furthermore, Sanger sequencing confirmed that the circREPS2 molecule was circular and formed by a back-splicing junction (Figure 1C). RNase R experiments showed that the level of the linear forms of REPS2 was obviously reduced after RNase R treatment, but RNase R could not digest circREPS2, indicating that circREPS2 possessed a closed-loop structure (Figure 1D). The quantitative real-time PCR results further showed that the expression levels of circREPS2 in 60 paired GC tissues were lower than those in adjacent normal tissues (Figure 1E). Additionally, clinical data demonstrated that low expression of circREPS2 was correlated with higher tumor-node-metastasis (TNM) stage, larger tumor size, and poor tumor differentiation in GC patients (Table 1). Consistent with the GC tissue samples data, the expression of circREPS2 was markedly lower in multiple GC cell lines (MKN-45, BGC-823, AGS, SGC-7901, MKN-28, and MGC-803) than in GES-1 cells (Figure 1F). Fluorescence *in situ* hybridization (FISH) analysis revealed that circREPS2 was predominantly located in the cytoplasm of BGC-823 cells and SGC-7901(Figure 1G). All these results indicated that circREPS2 levels were usually reduced in GC tissues and cell lines, suggesting that circREPS2 may be involved in GC progression.

**Table 1:** Correlations between circREPS2 expression and clinicopathological parameters in GC patients

Characteristics	Total	CircREPS2 expression		P value
	(N=60)	Low (%) n=44	High (%) n=16	
**Gender**				

Male	39	28 (71.8)	11 (28.2)	0.482
Female	21	16 (76.2)	5 (23.8)	

**Age (year)**				

<60	19	14 (73.7)	5 (26.3)	0.613
≥60	41	30 (73.2)	11 (26.8)	

**Differentiation grade**				

Good	31	19 (61.3)	12 (39.7)	0.0414^∗^
Poor	29	25 (86.2)	4 (13.8)	

**Tumor size**				

<5 cm	30	17 (56.7)	13 (43.3)	0.007^∗∗^
≥5 cm	30	27 (90)	3 (10)	

**Lymph node**				

N0	26	19 (73.1)	7 (26.9)	0.598
N1-N2	34	25 (73.5)	9 (26.5)	

**TNM stage**				

1+2	25	13 (52)	12 (48)	0.002^∗∗^
3+4	35	31 (88.6)	4 (11.4)	

**Distant metastasis**				

M0	51	37 (77.5)	14 (22.5)	0.99
M1	9	7 (77.8)	2 (22.2)	

The TNM staging system is based on the tumor (T), the extent of spread to the lymph nodes (N), and the presence of metastasis (M) ^∗^P<0.05, ^∗∗^P<0.01.

### circREPS2 Suppressed the Proliferation, Colony Formation, and DNA Synthesis Abilities of GC Cells

**Figure 2:**
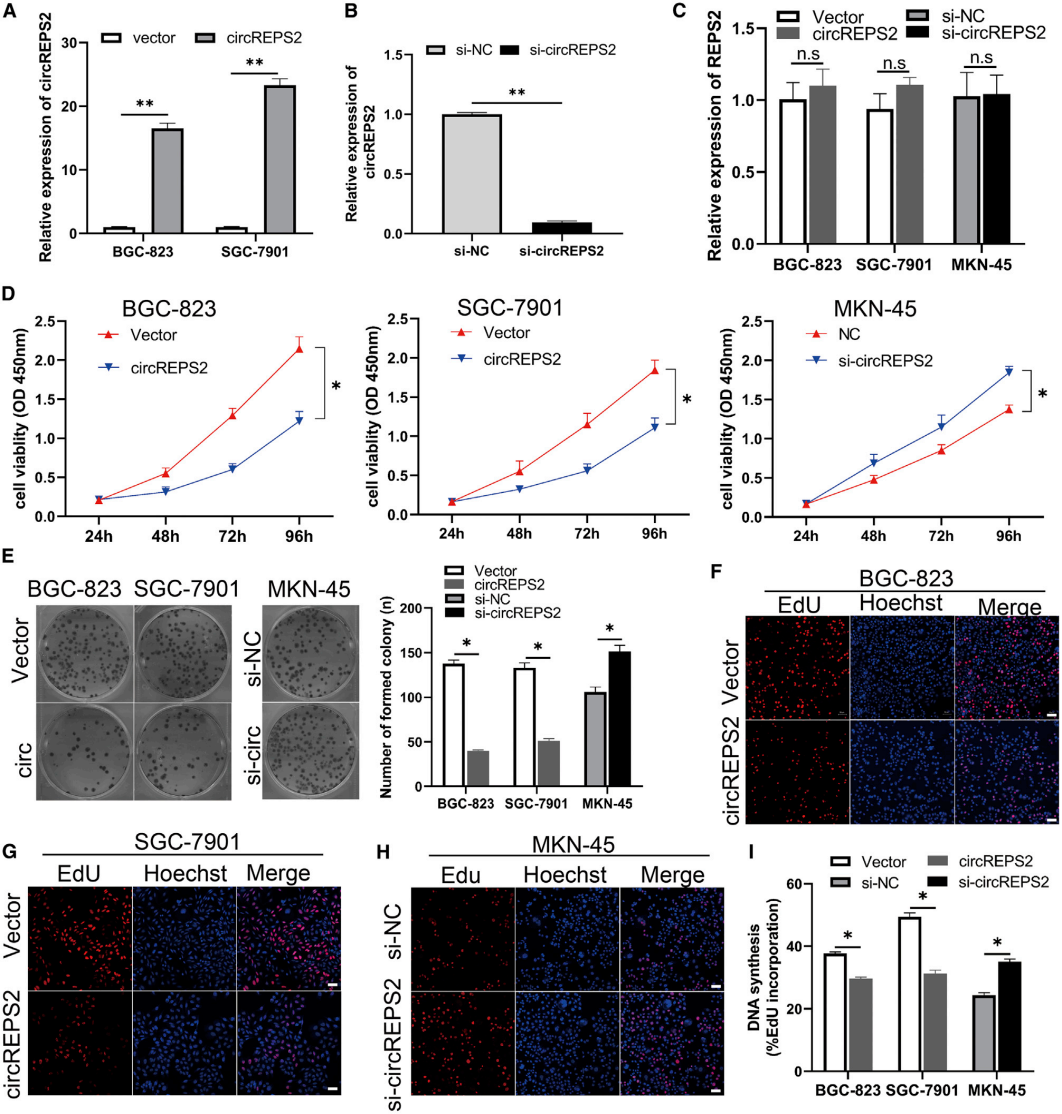
circREPS2 Suppressed the Proliferation, Colony Formation, and DNA Synthesis Abilities of GC Cells (A and B) Quantitative real-time PCR experiment confirmed that circREPS2 was successfully overexpressed in BGC-823 and SGC-7901 cell lines (A) and knocked down in MKN-45 cell line (B). (C) The expression of linear host gene REPS2 in in BGC-823, SGC-7901, and MKN-45 cells accessed by quantitative real-time PCR. (D and E) CCK-8 (D) and colony-formation assay (E) analysis of cell proliferation viability after transfection of the circREPS2 overexpression vector in BGC-823 and SGC-7901 and si-circREPS2 in MKN-45 cells. (F–I) EdU assay detected positive stained cell percent when overexpressing or knocking down circREPS2 in BGC-823 (F and I), SGC-7901 (G and I), and MKN-45 (H and I), respectively (scale bar, 50 μm). Values are shown as the mean ± standard error of the mean based on three independent experiments. ∗p < 0.05, ∗∗p < 0.01.

To study the functional roles of circREPS2 in GC, we used the circREPS2-overexpressing vector and circREPS2 small interfering RNA (siRNA) in gain- and loss-of-function experiments. The vector group is the empty vector control group, circREPS2 group is an overexpressed circREPS2 group. After BGC-823 and SGC-7901 cells were transfected with the circREPS2 overexpression vector, the overexpression efficiency of circREPS2 was detected and analyzed by quantitative real-time PCR assays (Figure 2A). Additionally, after transfecting circREPS2 siRNA into MKN-45 cells for approximately 48 h, the silencing efficiency of circREPS2 was detected by quantitative real-time PCR assays (Figure 2B). Importantly, overexpression and silencing of circREPS2 in GC cells did not influence the expression of REPS2, which is the host gene of circREPS2 (Figure 2C). Cell counting kit-8 (CCK-8) assays revealed that circREPS2 overexpression inhibited the proliferation of BGC-823 and SGC-7901 cells, whereas silencing of circREPS2 promoted the viability of MKN-45 cells (Figure 2D). Similarly, colony-formation assays showed that overexpression of circREPS2 suppressed the colony-forming ability of BGC-823 and SGC-7901 cells, while silencing of circREPS2 increased the colony-forming capacity of MKN-45 cells (Figure 2E). Also, 5-ethynyl-2′-deoxyuridine (EdU) assays indicated that overexpression of circREPS2 inhibited the DNA synthesis ability of BGC-823 (Figure 2F) and SGC-7901 cells (Figure 2G), while silencing of circREPS2 enhanced the DNA synthesis of MKN-45 cells (Figure 2H). These findings indicated the essential functional roles of circREPS2 in the viability and proliferation of GC cells *in vitro*.

### circREPS2 Suppressed the Migration, Invasion, and EMT in GC Cells

**Figure 3:**
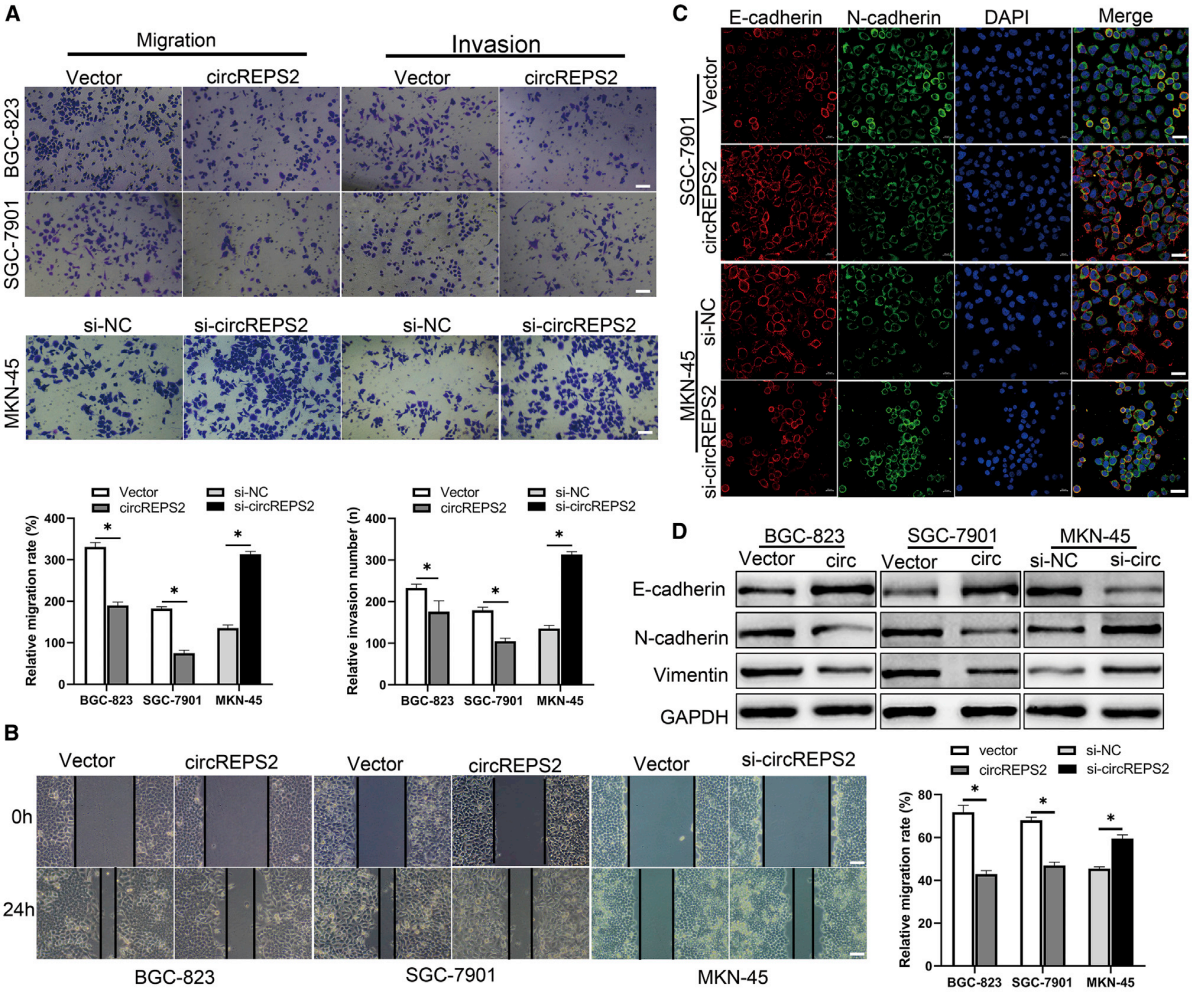
circREPS2 Suppressed the Migration, Invasion, and EMT of GC Cells (A) Transwell analysis of the migration and invasion potential after transfection of the circREPS2 expression vector or control vector in BGC-823 and SGC-7901 cells or si-circREPS2 or si-NC in MKN-45 cells (scale bar, 50 μm). (B) Wound-healing analysis of cell migration after overexpression of circREPS2 in BGC-823 and SGC-7901 cells, and silence circREPS2 in MKN-45 cells (scale bar, 10 μm). (C) IF analysis of the expression of E-cadherin and Vimentin after transfection of the circREPS2 expression vector or control vector in SGC-7901 cells or si-circREPS2 or si-NC in MKN-45 cells (scale bar, 40 μm). (D) Western blot analysis of the expression of EMT related markers after overexpression circREPS2 in SGC-7901 cells and knock down in MKN-45 cells. Values are shown as the mean ± standard error of the mean based on three independent experiments. ∗p < 0.05, ∗∗p < 0.01.

After investigating the roles of circREPS2 in proliferation, we further validated the ability of circREPS2 to affect migration and invasion. Transwell assays revealed that overexpression of circREPS2 prominently reduced the migration and invasion capabilities of BGC-823 and SGC-7901 cells. In contrast, the silencing of circREPS2 increased the migration and invasion capacities of MKN-45 cells compared to the si-normal control (NC) group (Figure 3A). Subsequently, wound-healing assays showed that overexpression of circREPS2 inhibited the migration of BGC-823 and SGC-7901 cells, while the migration of MKN-45 cells was dramatically elevated by si-circREPS2 (Figure 3B). Furthermore, immunofluorescence (IF) assays demonstrated that the expression of circREPS2 produced aggregated morphology and reduced the dispersion of SGC-7901 cells, accompanied by an increase in E-cadherin and a reduction in Vimentin. Additionally, the silencing of circREPS2 showed the opposite effects with elevated expression of Vimentin and decreased expression of E-cadherin in MKN-45 cells (Figure 3C). Western blotting showed similar changes in EMT-related markers, whereby overexpression of circREPS2 increased E-cadherin levels and decreased N-cadherin and Vimentin levels, and silence of circREPS2 reduced the expression of E-cadherin and upregulated the expression of N-cadherin and Vimentin (Figure 3D). These data further revealed that circREPS2 suppressed GC cell migration, invasion, and EMT *in vitro*.

### circREPS2 Functioned as a miR-558 Sponge in GC Cells

**Figure 4:**
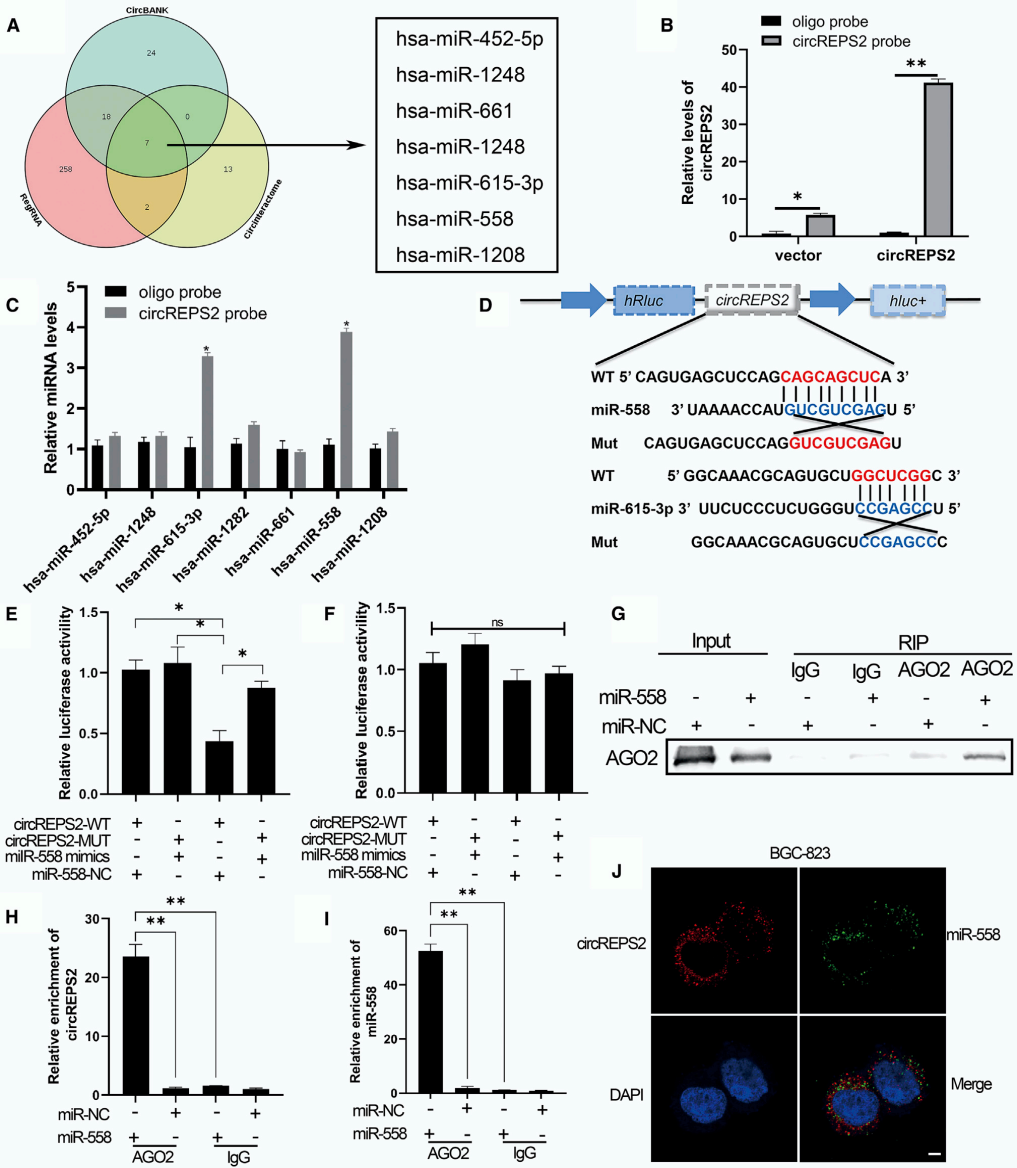
circREPS2 Functioned as a miR-558 Sponge in GC Cells (A) Schematic illustration of the overlapping target miRNAs of circREPS2 predicted by circBank, RegRNA, and CircInteractome. (B and C) RNA pull-down analysis of the enrichment of circREPS2 (B) and circREPS2-related 7 miRNA candidates in BGC-823 cells (C). (D) Schematic representation of potential binding sites of miR-558 and miR-615-3p with WT or MUT circREPS2. (E and F) The interaction between circREPS2 and miR-558 (E), miR-615-3p (F) as verified by dual-luciferase reporter assay. (G–I) Anti-AGO2 RIP analysis of the expression of AGO2 protein detected by western blotting (G), circREPS2 (H), and miR-558 (I) detected by quantitative real-time PCR in BGC-823 cells. (J) FISH analysis of the cellular colocalization of circREPS2 and miR-558 in BGC-823. Nuclei were stained with DAPI (scale bar, 20 μm). Values are shown as the mean ± standard error of the mean based on three independent experiments. ∗p < 0.05, ∗∗p < 0.01.

Multiple studies have confirmed that circRNA functions as a miRNA sponge.^13^ Our study then explored whether circREPS2 regulated the behavior of GC cells by sponging multiple miRNAs. First, through bioinformatics analysis, we identified seven miRNAs from the overlap of three databases (circBank,^14^ RegRNA,^15^ and CircInteractome^16^) as possible targets for circREPS2 (Figure 4A). To confirm those findings, we performed RNA pull-down assays and designed the specific circREPS2 probes. The results showed that circREPS2 in GC cells was clearly pulled down by the circREPS2 probe (Figure 4B). Two predicted miRNAs (miR-558 and miR-615-3p) with markedly increased fold changes in circREPS2 capture compared with the control probe were validated by quantitative real-time PCR (Figure 4C). Next, the full-length sequences of circREPS2-wild-type (WT) and circREPS2-mutant (MUT) without miR-558 or miR-615-3p binding sites were cloned into the luciferase vector (Figure 4D). Subsequently, luciferase reporter assays confirmed that miR-558 mimics markedly reduced the luciferase activity of circREPS2-WT but not the luciferase activity of circREPS2-MUT compared to the miR-NC group (Figure 4E). The miR-615-3p mimics did not show any significant difference not only in circREPS2-WT luciferase activity but also in circREPS2-MUT luciferase activity (Figure 4F). Therefore, we further focused on miR-558 as a downstream molecule of circREPS2. To further verify the relationship between circREPS2 and miR-558 in GC cells, anti-AGO2 RNA immunoprecipitation (RIP) assays revealed that, compared to immunoglobulin G (IgG), the anti-AGO2 antibody could efficiently pull down AGO2 in BGC-823 cells transfected with miR-558 mimics (Figure 4G). Quantitative real-time PCR validated the observation that circREPS2 and miR-558 were more enriched in the miR-558 mimic-transfected BGC-823 cell group than in the miR-NC group (Figures 4H and 4I). Based on the above results, we decided to conduct FISH assays, and the results showed that miR-558 and circREPS2 were colocalized in the cytoplasm of BGC-823 cells (Figure 4J). These results suggested that circREPS2 may function as a miR-558 sponge in GC cells.

### miR-558 Reversed the Ability of circREPS2 to Inhibit GC Cell Proliferation, Migration, Invasion, and EMT

**Figure 5:**
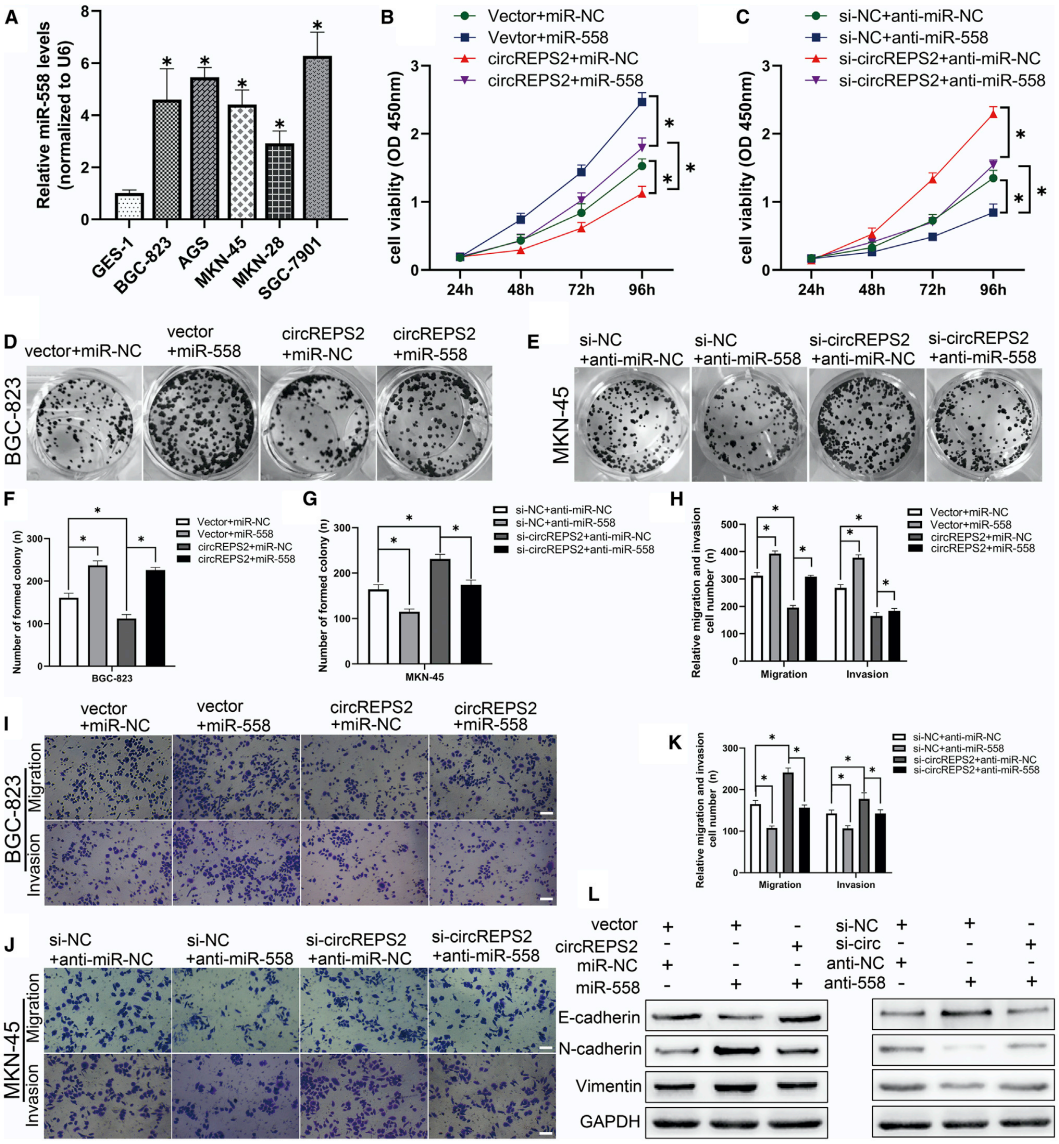
miR-558 Reversed circREPS2-Induced Decrease in Cell Proliferation, Migration, and Invasion in GC Cells (A) Quantitative real-time PCR analysis of miR-558 expression in various human GC cell lines (BGC-823, AGS, MKN-45, MKN-28, and SGC-7901) and GES-1. (B and C) CCK-8 analysis of cell viability after the cotransfection of miR-558 mimics and circREPS2 in BGC-823 cell (B) or miR-558 inhibitor and si-circREPS2 in MKN-45 cells (C). (D–G) Colony-formation analysis of colony numbers after the cotransfection of miR-558 mimics and circREPS2 in BGC-823 cells (D and F) or miR-558 inhibitor and si-circREPS2 in MKN-45 cells (E and G). (H–K) Transwell analysis of the cell migration potential after the cotransfection of miR-558 mimics and circREPS2 in BGC-823 cells (H and I) or miR-558 inhibitor and si-circREPS2 in MKN-45 cells (K and J) (scale bar, 50 μm). (L) Western blot analysis of the expression of E-cadherin, N-cadherin, and Vimentin after the cotransfection of miR-558 mimics and circREPS2 in BGC-823 cells or miR-558 inhibitor and si-circREPS2 in MKN-45 cells. Values are shown as the mean ± standard error of the mean based on three independent experiments. ∗p < 0.05, ∗∗p < 0.01.

To study the functional interaction of circREPS2 and miR-558 in GC cells, we performed rescue experiments in BGC-823 and MKN-45 cell lines. Quantitative real-time PCR analysis indicated that miR-558 levels were strikingly increased in GC cell lines (BGC-823, AGS, MKN-45, MKN-28, and SGC-7901) compared to GES-1 cells (Figure 5A). CCK-8 (Figures 5B and 5C) and colony-formation assays proved that the exogenous upregulation of miR-558 expression facilitated cell proliferation and colony formation in BGC-823 cells (Figures 5D and 5F), while downregulation of miR-558 had the opposite effects in MKN-45 cells (Figures 5E and 5G). Additionally, miR-558 mimics could partly reverse BGC-823 cell viability and colony-forming abilities suppressed by circREPS2 overexpression, and miR-558 inhibitor in part reversed the MKN-45 cell proliferation and colony-forming abilities promoted by si-circREPS2. Transwell assays demonstrated that miR-558 mimics improved the migration capabilities of BGC-823 cells (Figures 5H and 5I), whereas miR-558 inhibitor showed the reverse effects in MKN-45 cells. miR-558 mimics partly reversed BGC-823 cell migration inhibited by circREPS2 overexpression, and miR-558 inhibitor could in part reverse MKN-45 cell migration induced by si-circREPS2 (Figures 5J and 5K). Next, western blotting revealed that miR-558 mimics transfected into BGC-823 cells increased the Vimentin and N-cadherin protein levels and reduced the E-cadherin levels, and miR-558 inhibitor transfected into MKN-45 cells had the opposite effects. Similarly, miR-558 mimics partially reversed the EMT process inhibited by circREPS2 overexpression, and miR-558 inhibitor in part reversed the EMT process increased by si-circREPS2, accompanied by changes in the E-cadherin, N-cadherin, and Vimentin levels (Figure 5L). These data indicated that circREPS2 suppressed GC progression and metastasis partly by abrogating the tumor-promoting functional roles of miR-558.

### circREPS2 Regulated RUNX3 by Directly Targeting miR-558 in GC Cells

**Figure 6:**
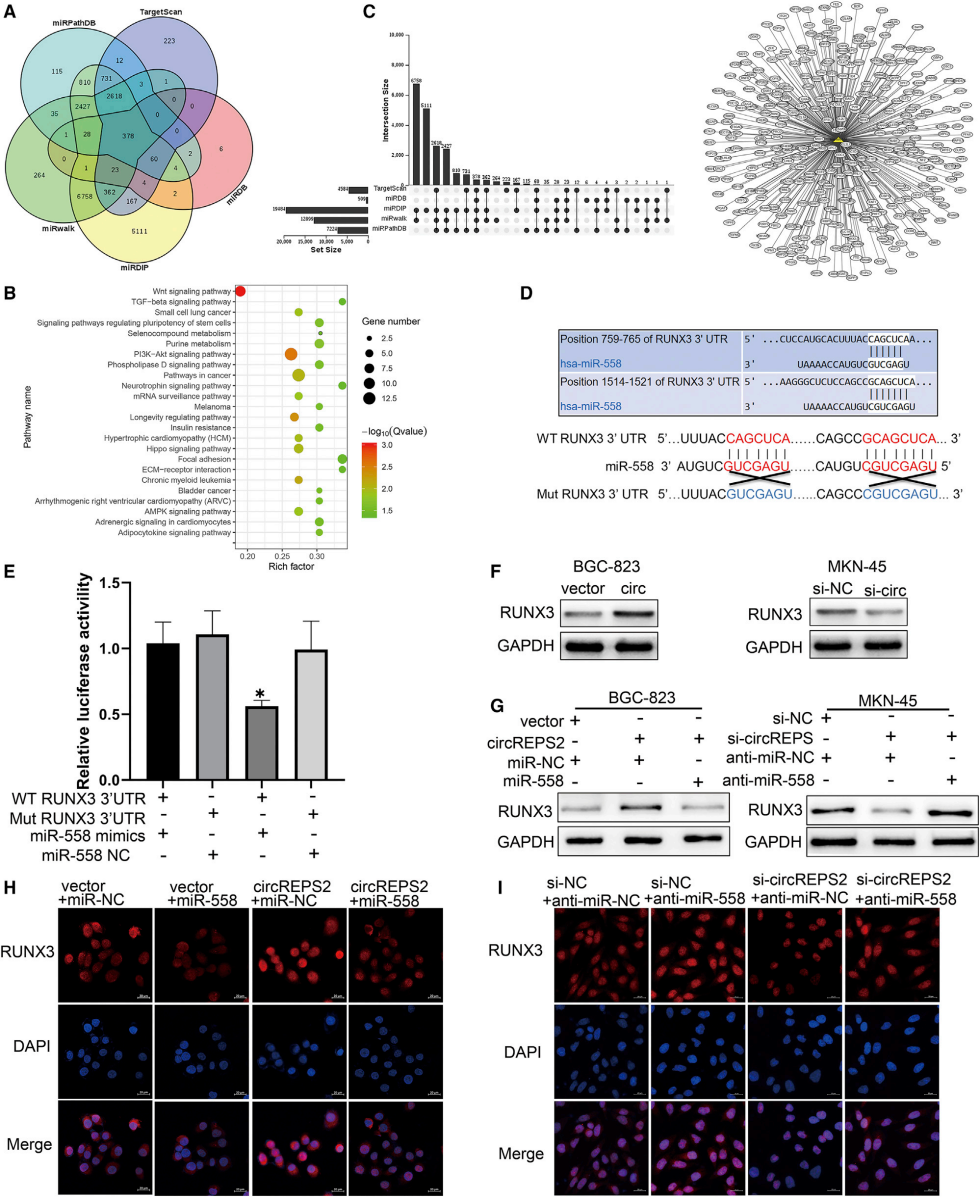
circREPS2 Binds to miR-558 and Thus Upregulates the Expression of RUNX3 (A) The databases were used to predict the target genes of miR-558. (B) KEGG enrichment analysis of the association of miR-558 expression with enriched signaling pathways. (C) Integration and visualization analysis of the 378 overlapping genes of miR-558. (D) Schematic representation of the potential binding sites of miR-558 in the WT or MUT RUNX3 3′ UTR. (E) The interaction between miR-558 and RUNX3 as verified by dual-luciferase reporter assay. (F) The expression of RUNX3 after overexpression and silencing of circREPS2. (G) Western blot analysis of the expression of RUNX3 after the cotransfection of circREPS2 and miR-558 mimics in BGC-823 cells or si-circPEPS2 and miR-558 inhibitor in MKN-45 cells. (H and I) IF analysis of the expression of RUNX3 after transfection of the circREPS2 expression vector and miR-558 mimics in (H) BGC-823 cells or si-circPEPS2 and miR-558 inhibitor in (I) MKN-45 cells (scale bar, 20 μm). Values are shown as the mean ± standard error of the mean based on three independent experiments. ∗p < 0.05, ∗∗p < 0.01.

In an attempt to determine which target genes are regulated by the circREPS2/miR-558 axis, bioinformatics analysis revealed that 378 predicted target genes of miR-558 were screened and validated by miRNA prediction tools, including the miRPathDB,^17^ TargetScan,^18^ miRwalk,^19^ miRDB,^20^ and miRDIP^21^ databases (Figure 6A). The Kyoto Encyclopedia of Genes and Genomes (KEGG) enrichment analysis using the KOBAS 3.0 online database indicated that 378 downstream genes of miR-558 were preferentially enriched in the Wnt/β-catenin pathway (Figure 6B). Additionally, the overlapping genes of the different prediction tools were integrated and visualized by TBtools and Cytoscape 3.6 software^22^ (Figure 6C). We then focused on one of the target genes, RUNX3, a tumor suppressor that can protect gastric epithelial cells against EMT,^23^ participates in the Wnt/β-catenin pathway,^24^ and contains two miR-558 binding sites. To confirm this prediction, we subcloned the sequences of the RUNX3 3′ UTR-WT and -MUT (without miR-558 binding sites) into the luciferase vector to perform the luciferase reporter assay (Figure 6D). The results showed that the luciferase activity of RUNX3 3′ UTR-WT was markedly reduced by miR-558 mimics in HEK293T cells, while that of RUNX3 3′ UTR-MUT was unchanged by miR-558 mimics compared with the control group (Figure 6E). Moreover, overexpression of circREPS2 markedly increased the expression levels of RUNX3 in BGC-823 cells, while silencing circREPS2 significantly reduced the levels of RUNX3 in MKN-45 cells (Figure 6F). Moreover, the upregulation or downregulation of RUNX3 caused by circREPS2 overexpression or silencing was reversed by miR-558 mimics or inhibitor, respectively (Figure 6G). Similarly, IF assays also showed the same results in BGC-823 (Figure 6H) and MKN-45 cells (Figure 6I), confirming that RUNX3 expression may be regulated by the circREPS2/miR-558 axis. These data further demonstrated that circREPS2 regulated RUNX3 levels by sponging miR-558 in GC.

### circREPS2 Suppressed GC Cell Viability, Migration, Invasion, and EMT by Sponging miR-558 and Upregulating RUNX3 Expression to Inactivate β-catenin Signaling

**Figure 7:**
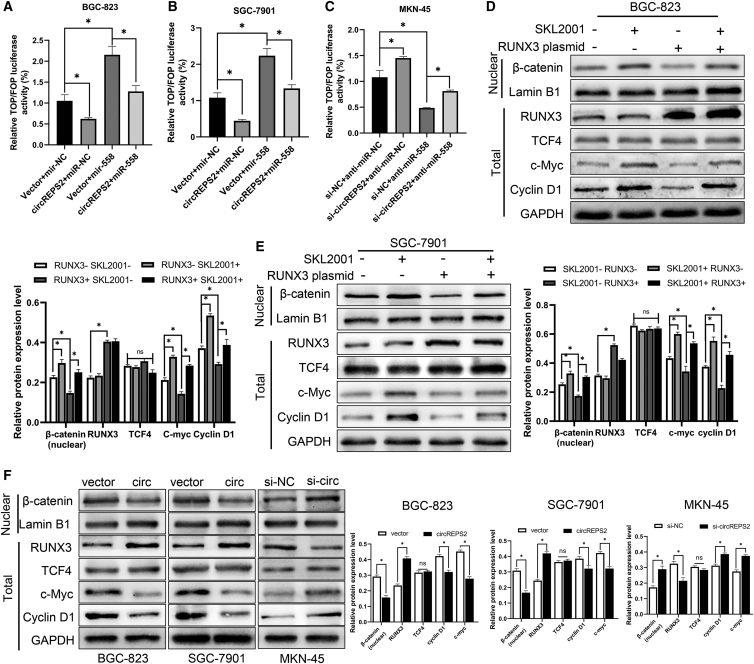
circREPS2 Inhibits miR-558 Upregulating RUNX3 Expression, thereby Inhibiting Wnt/β-catenin Pathway Transcriptional Activity (A and B) TOP/FOP luciferase activity of BGC-823 (A) and SGC-7901 (B) cells after transfection with miR-NC, miR-558 mimics, circREPS2 overexpression vector, and miR-558 mimics + circREPS2 overexpression vector. (C) TOP/FOP luciferase activity of MKN-45 cells after transfection with miR-NC, miR-558 inhibitor, si-circREPS2, and miR-558 inhibitor + si-circREPS2. (D and E) Western blotting analysis of nuclear β-catenin, RUNX3, TCF4, c-Myc, and Cyclin D1 protein levels in BGC-823 cells (D) and SGC-7901 cells (E) after transfection with NC, SKL200, RUNX3 plasmid, and SKL200 + RUNX3 plasmid. (F) Western blotting analysis of overexpression circREPS2 in BGC-823, SGC-7901, and silencing circREPS2 in MKN-45 cells, RUNX3, and β-catenin pathway related protein expression. Values are shown as the mean ± standard error of the mean based on three independent experiments. ∗p < 0.05, ∗∗p < 0.01.

Based on the prediction that Wnt/β-catenin signaling is downstream, we performed a series of experiments to validate whether the circREPS2/miR-558 axis could regulate β-catenin signaling in GC. TOP/FOP reporter assays were performed in GC cells. TOP/FOP luciferase activity was increased or decreased in BGC-823 (Figure 7A), SGC-7901 (Figure 7B), and MKN-45 (Figure 7C) cells after transfection with the circREPS2 overexpression plasmid or si-circREPS2, respectively. Further investigations indicated that miR-558 mimics could reverse the decrease in TOP/FOP luciferase activity induced by circREPS2 overexpression in BGC-823 and SGC-7901 cells (Figures 7A and 7B). Accordingly, the miR-558 inhibitor reversed the increase in TOP/FOP luciferase activity induced by si-circREPS2 in MKN-45 cells (Figure 7C). In addition, the Wnt/β-catenin agonist SKL2001^25^ and the RUNX3 plasmid were transfected into SGC-7901 and BGC-823 cells. Western blotting confirmed that β-catenin signaling was activated after GC cells were cultured with SKL2001, accompanied by the enhanced expression of the downstream molecules Cyclin D1, c-Myc, and nuclear β-catenin in BGC-823 cells (Figure 7D) and SGC-7901(Figure 7E). When we used the RUNX3 plasmid to exogenously increase the expression of RUNX3, the Wnt/β-catenin pathway was significantly inhibited in both BGC-823 (Figure 7D) and SGC-7901 cells (Figure 7E), resulting in a significant decrease in nuclear β-catenin and downstream target genes Cyclin D1, c-Myc. Conversely, when cells were co-transfected with SKL2001 and RUNX3 plasmids, the SKL2001 partially reversed the inhibition of Wnt/β-catenin caused by RUNX3, resulting in the recovery of nuclear b-catenin, c-Myc, and Cyclin D1 expression. These results indicate that RUNX3 can significantly inhibit the transcriptional activity of Wnt/β-catenin signal pathway. Then, we overexpressed circREPS2 in BGC-823, SGC-7901, and knocked down circREPS2 in MKN-45 cell lines, respectively, to explore the effect on Wnt/β-catenin. Wb results showed that overexpression of circREPS2 could lead to increased expression of RUNX3, which resulted in inhibition of Wnt/β-catenin and decreased expression of β-catenin in the nucleus and downstream target genes Cyclin D1 and c-Myc. Conversely, knocking down circREPS2 in MKN-45 cells produced the opposite result (Figure 7F). These results suggest that circREPS2 may inhibit the transcriptional activity of β-catenin by promoting the expression of RUNX3.

### The Effects of circREPS2 on Tumor Growth *In Vivo*

**Figure 8:**
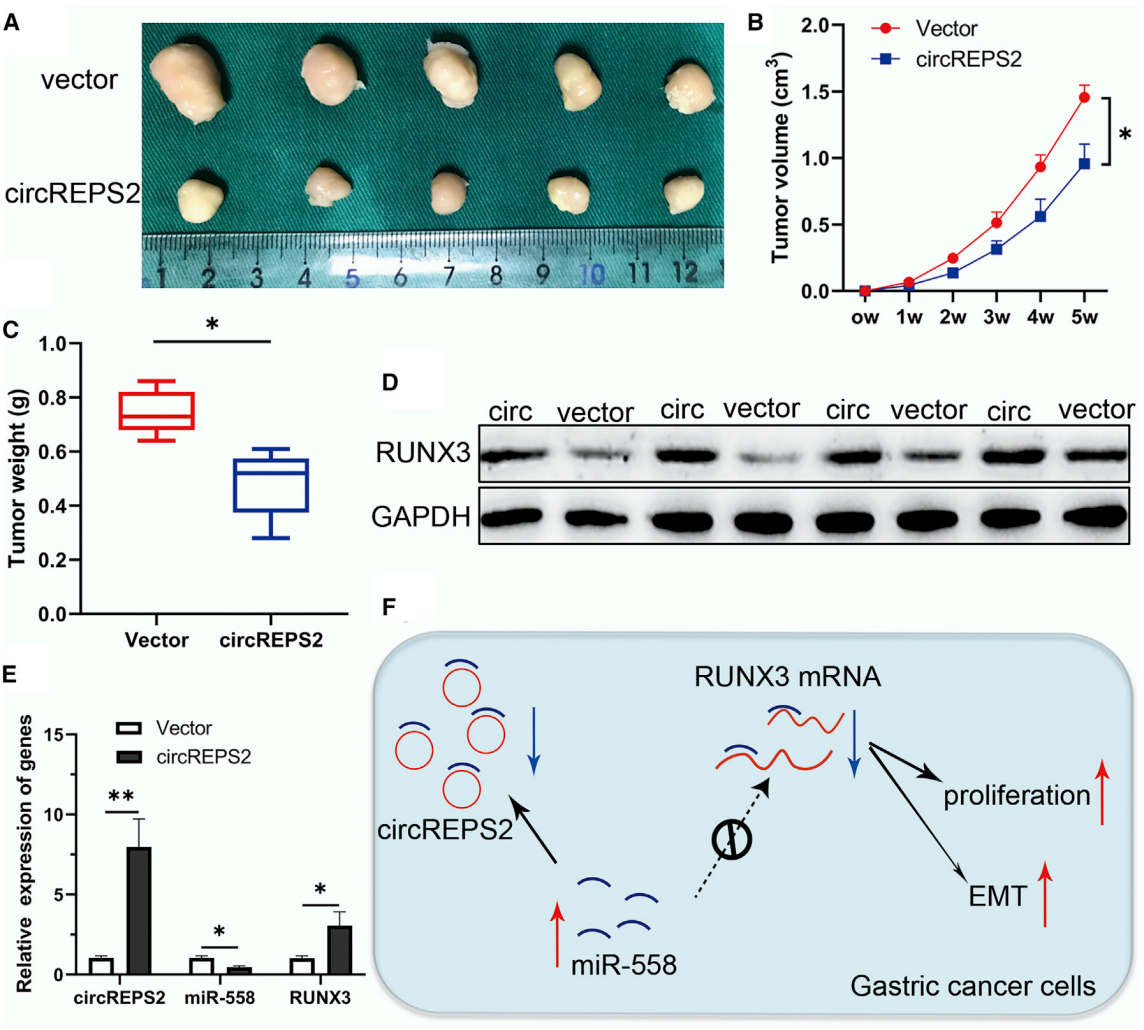
The Effects of circREPS2 on Tumor Growth *In Vivo* (A) Comparison of the tumor size of xenograft tumors induced by circREPS2 overexpression and NC vector in transfected SGC-7901 cells. (B) A growth curve analysis of tumor growth in SGC-7901 cells transfected with the circREPS2 overexpression vector or NC vector. (C) Comparison of the tumor weight in SGC-7901 cells transfected with the circREPS2 overexpression vector and NC vector. (D) The protein level of RUNX3 was detected by western blot. (E) The mRNA level of circREPS2, miR-558, and RUNX3 were detected by quantitative real-time PCR. (F) Schematic representation of the circREPS2 mechanism in GC cells. Values are shown as the mean ± standard error of the mean based on three independent experiments. ∗p < 0.05, ∗∗p < 0.01.

To clarify whether circREPS2 influenced tumor growth *in vivo*, a circREPS2-overexpressing vector or a NC vector was stably transfected into GC cells. Xenograft tumor assays indicated that the xenograft tumors caused by circREPS2 overexpression were smaller than those caused by the NC vector (Figure 8A). The volume and weight of tumors were markedly reduced in the circREPS2 overexpression group compared with the NC vector group (Figures 8B and 8C). Consistent with the *in vitro* observations, the circREPS2-overexpression group displayed upregulated protein levels of RUNX3 (Figure 8D). Additionally, overexpression of circREPS2 triggered an increase of circREPS2 and RUNX3 mRNA level while a reduction of miR-558 in excised tumor masses (Figure 8E). Overall, these findings demonstrated that circREPS2 sponged miR-558 and upregulated RUNX3 to inactivate β-catenin signaling, thereby inhibiting the progression and metastasis of GC (Figure 8F).

## Discussion

Mounting evidence has demonstrated that circRNAs are not only potential biomarkers and therapeutic targets for multiple cancers,^26^ but also act as carcinogenic or tumor-suppressive molecules in a variety of diseases.^27^ For example, circPICALM is confirmed as a definite prognostic factor in patients with bladder cancer.^28^ Here, our data showed that circREPS2 levels were not only lower in GC tissues compared to noncancerous tissues but also in GC cell lines compared to GES-1 cells. Additionally, low expression of circREPS2 was associated with a higher TNM stage, poor tumor differentiation, and larger tumor size in GC patients. Next, functional experiments indicated that overexpression of circREPS2 in GC cells inhibited proliferation, migration, EMT, and invasion *in vitro* and tumor growth *in vivo*, whereas silencing of circREPS2 had the opposite effects. This finding demonstrated that circREPS2 might be a novel promising biomarker and inhibitory molecule in GC cells.

Many studies have indicated that the primary function of circRNAs is to act as miRNA sponges to regulate gene expression, thereby affecting tumor progression. For instance, circ_0078767 regulates RASSF1A expression by sponging miR-330-3p to suppress the proliferation, invasion, and migration of lung cancer cells.^29^ Our team previously reported that circGRAMD1B inhibits GC cell proliferation, invasion, and migration by binding miR-130a-3p and regulating PTEN/p21 signaling.^12^ Here, RIP, FISH, luciferase reporter assay, and RNA pull-down analysis showed that circREPS2 was confirmed to function as a miR-558 sponge in GC cells. Rescue experiments also showed that miR-558 was increased in GC cells and reversed the anticancer effect of circREPS2 in GC cells. Furthermore, bioinformatics, luciferase reporter, western blot, and IF analyses proved that RUNX3 was a downstream target gene of miR-558 and an indirect regulatory gene of circREPS2 in GC cells. These results indicated that circREPS2 could directly sponge miR-558 and indirectly regulate RUNX3 to suppress the progression and metastasis of GC cells.

EMT, which is characterized by the loss of epithelial features and the acquisition of mesenchymal features, is a key step in cancer metastasis.^30^^,^^31^ The markers of EMT in GC are associated with poor patient outcomes.^32^ Moreover, emerging evidence suggests that the Wnt/β-catenin pathway can induce EMT.^33^ Here, the results of the TOP/FOP luciferase assays showed that β-catenin signaling was activated/suppressed by the miR-558 mimic/inhibitor. Cotransfection with si-circREPS2/circREPS2-overexpressing vector and miR-558 inhibitor/mimics increased or decreased TOP/FOP activity. Western blot shown than RUNX3 overexpression plasmid can reverse the activation of the Wnt/β-catenin pathway caused by SKL2001, resulting in decreased expression of nuclear β-catenin, c-Myc, and Cyclin D1. The results demonstrated that increased expression of RUNX3 inhibited β-catenin signaling in GC. Additionally, it has been reported that RUNX3 reduces β-catenin nuclear localization, transactivation, and protein stability in GC cells.^34^ Our results suggest that RUNX3 may have the same effect in GC cells. In addition, RUNX3 has been reported to have an additional inhibitory effect on the Wnt/β-catenin pathway. It can suppress the transcriptional activity of the Wnt/β-catenin pathway by binding to the TCF4 and β-catenin protein complex in intestinal tumorigenesis.^35^ In short, our findings indicate that circREPS2 acts as a miR-558 sponge and upregulates RUNX3, thus affecting the transcriptional activity of the β-catenin pathway and inhibiting GC proliferation, migration, invasion, and EMT.

Taken together, our study demonstrated that circREPS2 expression is significantly reduced in GC tissues and that low levels of circREPS2 expression correlate with higher TNM stage, poor tumor differentiation, and larger tumor size in GC patients. Mechanistically and functionally, circREPS2 suppresses GC cell proliferation, metastasis, and EMT by sponging miR-558 and upregulating RUNX3 expression. Based on our data, we suggest that circREPS2 could be a therapeutic target for GC patients. Moreover, the circREPS2/miR-558/RUNX3/β-catenin regulatory network provides a novel therapeutic target for the management of GC.

## Materials and Methods

### Clinical Samples

All GC tissues and matched noncancerous tissues from primary GC patients were obtained from the First Affiliated Hospital of Chongqing Medical University between 2014 and 2019. No patients received local or systemic preoperative chemotherapy or radiotherapy, and all tissues were histologically identified by two pathologists. The resected specimens were processed immediately and stored at −80°C. Moreover, the circRNA microarray of five paired human GC tissues was constructed and completed at Beijing CapitalBio Technology (Beijing, China). This study was approved by the Ethics Committee of Chongqing Medical University and was in compliance with the relevant guidelines. Informed consent was obtained from all GC participants.

### Cell Culture

Human GC cell lines (AGS, BGC-823, SGC-7901, MKN-28, MKN-45, and MGC-803), a human embryonic kidney cell line (HEK293T), and a human gastric epithelial cell line (GES-1) were purchased from the Type Culture Collection of the Chinese Academy of Sciences (Beijing, China). Cell lines were cultured in DMEM (HyClone, Shanghai, China) or RPMI 1640 (GIBCO, Carlsbad, USA) containing 10% fetal bovine serum (FBS, Biological Industry, Israel). Cell lines were maintained and cultured in a cell incubator at 37°C with 5% CO_2_.

### RNA Preparation, Quantitative Real-Time PCR, and RNase R Treatment

Total RNA was obtained using TRIzol reagent (Takara, Japan) following the manufacturer’s protocol. Then, reverse transcription was performed by PrimeScript RT Reagent Kit (Takara, #RR037A), and quantitative real-time PCR was carried out using TB Green Premix Ex TaqII (Takara, #RR820A). The divergent primer for circREPS2 was designed by Geneseed (Guangzhou, China), and the other primers were designed and synthesized by RiboBio (Guangzhou, China) or Sangon Biotech (Shanghai, China). The sequences of the primers used for quantitative real-time PCR are listed in Table S1. The 2^−ΔΔCt^ method was used to calculate the relative expression levels. For RNase treatment, 2 μg of total RNA was incubated with or without 3 U/μg RNase R for 30 min at 37°C.

### Plasmid Construction and Oligonucleotide Transfection

To establish the circREPS2 stably overexpressing cell lines, we cloned a 489 bp cDNA of the circREPS2 sequence into the pHBLV-CMV-crRNA plasmid lentiviral vector obtained from Hanbio Biotech (Shanghai, China). The transfected cells were then selected with puromycin (2 μg/mL). circREPS2-specific siRNA was designed and purchased by GenePharma (Shanghai, China). miRNA mimic, inhibitor, and its normal control were purchased from RiboBio (Guangzhou, China). The miRNA primer sequences are reasonably needed and can be provided by them. WT and MUT circREPS2 and RUNX3 3′ UTR plasmids were designed and purchased from Gene Create (Wuhan, China). The nucleotide sequences are listed in Table S1. Lipofectamine 2000 (Invitrogen, Carlsbad, USA) was used for plasmid and oligonucleotide transfection in accordance with the manufacturer’s guidelines.

### FISH

Cy3-labeled circREPS2 probes and FAM-labeled miR-558 probes (RiboBio, China) were detected in GC cells using a Fluorescent *In Situ* Hybridization Kit (RiboBio, China) following the manufacturer’s guidelines. In brief, the probes specific to circREPS2 and miR-558 were hybridized overnight. Next, cell nuclei were counterstained with DAPI (Beyotime, China). The glass slides were analyzed and images were captured under a ZEISS LSM800 fluorescence microscope (Carl Zeiss AG, Germany). The sequences of the circREPS2 and miR-558 probes are listed in Table S1.

### Cell Proliferation and Colony-Formation Assays

For the cell proliferation assay, a total of 10^3^ transfected GC cells/well were maintained in 96-well plates. Next, at 0, 24, 48, 72, and 96 h post treatment, 10 μL CCK-8 reagent was added to each well, and the cells were incubated for 2 h. The absorbance of the wells was measured at 450 nm spectrophotometrically. For the colony-formation assay, 24-well plates were used to seed 200 transfected GC cells/well using complete medium, and then cells were cultured in the incubator for 12 days. The cells were subsequently fixed with methanol and stained with crystal violet, and then colonies were imaged and counted.

### EdU Staining

Treated GC cells were seeded in 96-well plates (10^3^ cells/well) and cultured to a logarithmic growth phase. DNA synthesis and cell viability were measured and evaluated by a Cell-Light EdU DNA Cell Proliferation Kit (RiboBio, Guangzhou, China) in accordance with the manufacturer’s protocol. Briefly, 4% formalin and 0.5% Triton X-100 were used to fix and permeabilize GC cells, respectively. EdU was stained with Apollo reaction solution (100 μL), and cell nuclei were stained with Hoechst 33342 (100 μL). Then, the cells were detected and imaged by a ZEISS LSM800 confocal microscope (Carl Zeiss AG, Germany).

### Transwell and Wound-Healing Assays

Transfected GC cells (4 × 10^4^) were seeded in a Transwell chamber (Costar, USA) for the migration assay or in a chamber precoated with 100 μL of 1 μg/μL Matrigel matrix (BD Biosciences, USA) for the invasion assay. Serum-free medium and complete medium were added to the upper and lower chambers, respectively. After 24 h of incubation, cells were fixed with methanol and stained with crystal violet. After that, the cells were observed under a microscope. For the wound healing assays, transfected GC cells were cultured in a 6-well plate to 90% confluence. A 10 μL sterile pipette tip was used to create thin scratches with the same width. Images were acquired instantly (0 h) using a microscope. The width of the wounds was recorded (24 h to 48 h) again after incubation in serum-free medium.

### RNA Pull-Down Assay

The circREPS2 probe and oligo probe were designed by GenePharma (Shanghai, China), and pull-down assays were carried out as described.^12^ In short, approximately 1 × 10^7^ BGC-823 cells were sonicated and lysed to obtain cell lysates. The probes were incubated with M280 streptavidin Dynabeads (Invitrogen) to generate probe-coated beads, which were incubated with the cell lysates at 4°C overnight. Then, the RNA mixture bound to the beads was washed, extracted with TRIzol Reagent, and used for analysis.

### RIP Binding Protein

The Magna RIP Kit (Millipore, USA) was used to perform and analyze RIP assays. In brief, approximately 2 × 10^7^ BGC-823 cells were transfected with miR-558 mimics or miR-NC using Lipofectamine 2000. The treated cells were resuspended and lysed in RIP lysis buffer. Cell lysates were incubated with RIP immunoprecipitation buffer containing magnetic beads conjugated with anti-Ago2 antibody (Cell Signaling Technology, USA) and control IgG antibody at 4°C overnight. After incubation with Proteinase K, the immunoprecipitated RNA was eluted, isolated, and quantified by quantitative real-time PCR and western blotting.

### Dual-Luciferase Reporter and TOP/Flash and FOP/Flash Reporter Assays

The WT or MUT plasmid (pGL3-Firefly_Luciferase-Renilla_Luciferase) of circREPS2 and the RUNX3 3′ UTR were designed and constructed by Gene Create (Wuhan, China). HEK293T cells were cotransfected with the plasmid and miR-558 mimics, miR-615-3p, or miR-NC using Lipofectamine 2000. After incubation for 48 h, the Dual-Luciferase Assay Kit (Beyotime, China) was applied to perform the luciferase reporter experiments. Next, GC cells were cultured and cotransfected with miR-NC, miR-558 mimics, anti-miR-558, miR-558 mimics + circREPS2 overexpression vector, and anti-miR-558 + circREPS2 siRNA combined with TOPFlash, FOPFlash, and Renilla luciferase plasmids (Addgene, Cambridge, USA). After transfection for 48 h, GC cells were lysed and stimulated with 15% Wnt3A- or 20 mM LiCl-containing medium. All relative luciferase activities were quantified and analyzed with the Luciferase Reporter Gene Assay Kit (Promega).

### Agonist of β-catenin Signaling and RUNX3 Plasmid Construction

SKL2001, purchased from Med MedChemExpress (MCE, China), is a novel agonist of Wnt/β-catenin signaling and increases β-catenin transcription by elevating the levels of β-catenin in cells.^25^ To observe the effects of RUNX3 on the Wnt/β-catenin pathway, we constructed a RUNX3 overexpression plasmid (LAB cell, Chongqing, China). Untransfected cells or cells transfected with the RUNX3 plasmid were cultured in SKL2001 (40 μM) conditioned medium, and then the Wnt/β-catenin pathway-related protein levels were assessed by western blot.

### Western Blotting and IF

Briefly, proteins were prepared by radioimmunoprecipitation assay (RIPA) buffer and quantified by a BCA Protein Assay Kit (Beyotime, China). The nuclear protein was obtained using Nuclear and Cytoplasmic Protein Extraction Kit (Beyotime) following the manufacturer’s protocol. Samples were subjected to 10% SDS-PAGE and then transferred to polyvinylidene fluoride (PVDF) membranes (Millipore). The membranes were blocked and then sequentially incubated with the primary antibody, the secondary antibody, and detected by chemiluminescence. The antibodies used included primary antibodies against RUNX3, E-cadherin, Vimentin, N-cadherin, β-catenin, c-Myc, CD44, CCND1, GAPDH (glyceraldehyde-3-phosphate dehydrogenase, as negative control), and β-actin (Proteintech, USA). IF assays were conducted as reported previously.^12^ In brief, cells grown on slides, including the transfected GC cells, were successively incubated with primary antibodies (against E-cadherin and Vimentin) and fluorescent secondary antibodies (Beyotime) and counterstained with 4,6-diamidino-2-phenylindole (DAPI) (nucleus). Cells on the slides were observed and imaged using a ZEISS LSM800 fluorescence microscope (Carl Zeiss AG, Germany).

### Animal Experiments

BALB/c nude mice (female, 4 weeks old, five mice per group) were purchased from the National Laboratory Animal Center (Shanghai, China). SGC-7901 cells were transfected with circREPS2 overexpression lentivirus or NC vector, and then the treated cells were subcutaneously injected into the dorsal flanks of BALB/c nude mice (2 × 10^6^ cells per mouse). Xenograft tumors were excised for further analysis 5 weeks later, and tumor size was detected every week. All animal experiments were approved by the Animal Ethics Committee of Chongqing Medical University.

### Statistical Analysis

All data are presented as the mean ± standard deviation (SD) of at least three independent experiments. Experimental results were analyzed using Student’s t test (two-tailed) or ANOVA with GraphPad Prism 8. The correlation between circREPS2 expression and clinicopathological characteristics was calculated and analyzed using the chi-square test (χ2) or Fisher’s exact test. The differences were deemed statistically significant with ∗p < 0.05 or ∗∗p < 0.01.

## Author Contributions

X.G. and X.D. designed this study, performed the statistical analysis, and drafted the manuscript; A.C. and C.Q. collected tissue samples and analyzed the clinical data; Z.W. and J.L. critically revised the work for important intellectual content; and Z.W. supervised the study. All authors read and gave final approval of the manuscript.

## Conflicts of Interest

The authors declare no competing interests.

## Supplemental Information

Document S1. Table S1

Document S2. Article plus Supplemental Information
