# Supplementary material for: Circular RNA circREPS2 Acts as a Sponge of miR-558 to Suppress Gastric Cancer Progression by Regulating RUNX3/β-catenin Signaling
Source: Mol Ther Nucleic Acids. 2020 Jun 27;21:577–91. doi: 10.1016/j.omtn.2020.06.026 (PMC7390859; doi:10.1016/j.omtn.2020.06.026)
Supplement: Document S1. Table S1 [file mmc1.pdf]

OMTN, Volume *21*

Supplemental Information

Circular RNA circREPS2 Acts as a Sponge of miR-558 to Suppress Gastric Cancer Progression by Regulating RUNX3/β-catenin Signaling

Xiong Guo, Xinglong Dai, Jianjun Liu, Anqi Cheng, Chuan Qin, and Ziwei Wang

Supplementary table S1 Primers and oligonucleotide sequences used in this study

**List of oligonucleotide sequences**	**5'--> 3'**
**Primers for PCR**	
GAPDH Forward	CAATGACCCCTTCATTGACC
GAPDH Reverse	TTGATTTTGGAGGGATCTCG
CircREPS2 Forward	TGGTTCAAGTGACACTACCA
CircREPS2 Reverse	TTGCTGGACTACTTTCTGCA
REPS2 Forward	CAGTCTCCCACGATGTCACC
REPS2 Reverse	TTGTTGATTTGCCGGCAGTG
RUNX3 Forward	AGGCAATGACGAGAACTACTCC
RUNX3 Reverse	CGAAGGTCGTTGAACCTGG
U6 Forward	CTCGCTTCGCCAGCACA
U6 Reverse	AACGCTTCACGAATTTGCGT
**siRNAs**	
si-NC	GTGACAGATGGTTCAGTCCTA
si-circREPS2	CACTGTGATGGTTCAGTCCTA
**FISH probes**	
circREPS2-cy3	CCTGACTTGGTAGTGTCACTTGAAC
miR-558-FAM	ATTTTGGTACAGCAGCTCA
**Biotinylated probes**	
biotin-NC	GTGTAACACGTCTATACGCCCA
biotin-REPS2	CATGG TTCAAGTGACACTACCAAGTCAGGAT
biotin-NC mimics sense	UUCUCCGAACGUGUCACGUTT
biotin-miR-558 mimics sense	UGAGCUGCUGUACCAAAAU
**miRNA mimics and inhibitors**	
mimics NC	UUCUCCGAACGUGUCACGUTT
miR-558 mimics	UGAGCUGCUGUACCAAAAU
Anti-miR-NC	UCUACUCUUUCUAGGAGGUUGUGA
Anti-miR-558	AUUUUGGUACAGCAGCUCA
